# Advancing Prone-Transpsoas Spine Surgery: A Narrative Review and Evolution of Indications with Representative Cases

**DOI:** 10.3390/jcm13041112

**Published:** 2024-02-16

**Authors:** Peter N. Drossopoulos, Anas Bardeesi, Timothy Y. Wang, Chuan-Ching Huang, Favour C. Ononogbu-uche, Khoi D. Than, Clifford Crutcher, Gabriel Pokorny, Christopher I. Shaffrey, John Pollina, William Taylor, Deb A. Bhowmick, Luiz Pimenta, Muhammad M. Abd-El-Barr

**Affiliations:** 1 Division of Spine, Department of Neurosurgery, Duke University, Durham, NC 27710, USAkhoi.than@duke.edu (K.D.T.);; 2 Institute of Spinal Pathology, Sao Paulo 04101000, SP, Brazil; g.pokorny@patologiadacoluna.com.br (G.P.);; 3 Department of Neurosurgery, Jacobs School of Medicine and Biomedical Sciences, University at Buffalo, Buffalo, NY 14203, USA; 4 Department of Neurological Surgery, University of California, La Jolla, San Diego, CA 92093, USA

**Keywords:** spine, fusion, prone-transpsoas, PTP, LLIF, deformity

## Abstract

The Prone Transpsoas (PTP) approach to lumbar spine surgery, emerging as an evolution of lateral lumbar interbody fusion (LLIF), offers significant advantages over traditional methods. PTP has demonstrated increased lumbar lordosis gains compared to LLIF, owing to the natural increase in lordosis afforded by prone positioning. Additionally, the prone position offers anatomical advantages, with shifts in the psoas muscle and lumbar plexus, reducing the likelihood of postoperative femoral plexopathy and moving critical peritoneal contents away from the approach. Furthermore, operative efficiency is a notable benefit of PTP. By eliminating the need for intraoperative position changes, PTP reduces surgical time, which in turn decreases the risk of complications and operative costs. Finally, its versatility extends to various lumbar pathologies, including degeneration, adjacent segment disease, and deformities. The growing body of evidence indicates that PTP is at least as safe as traditional approaches, with a potentially better complication profile. In this narrative review, we review the historical evolution of lateral interbody fusion, culminating in the prone transpsoas approach. We also describe several adjuncts of PTP, including robotics and radiation-reduction methods. Finally, we illustrate the versatility of PTP and its uses, ranging from ‘simple’ degenerative cases to complex deformity surgeries.

## 1. Historical Review

In 2006, Luiz Pimenta and William Taylor published a seminal manuscript describing the technical aspects of lateral lumbar interbody fusion (LLIF) [1]. At the time, accessing the disc space anteriorly through the retroperitoneal space was considered by some to be revolutionary. Others, however, doubted its ability to gain popularity. This was in part due to its unfamiliar set-up (lateral decubitus position), the need for proprietary equipment, potential injury to the lumbar plexus, and proximity to the kidney, bowel, and retroperitoneal great vessels. Indeed, the initial adoption of the LLIF was associated with a steep learning curve, and complications previously absent in traditional posterior-approach spine surgery began to surface. Beyond the foreseen complications, such as femoral nerve palsy, there were reports of bowel or peritoneal violation [2], ureteral and renal injury [3], aorta or vena cava laceration [4], and psoas hematoma [5]. Despite these initial setbacks, continued refinements in technique, technology, and indications have given this surgical approach the ability to effectively treat a wide variety of lumbar pathologies, including de novo degenerative disc disease, spondylolisthesis, calcified disc herniations, and adjacent segment disease. More recent developments in implant technology have introduced the ability to perform lateral lumbar corpectomy [6] and anterior column release [7], which expand the indication for the lateral approach to those with thoracolumbar trauma, deformity, or even spinal oncology [8]. Indeed, lateral lumbar fusion is now an internationally recognized approach to the lumbar spine.

Despite its increasing adoption and excellent outcomes, there are still several drawbacks to the lateral lumbar interbody fusion that require attention when screening patients for intervention. For example, the traditional lateral lumbar interbody fusion is unable to access the L5-S1 disc space due to the positioning of the iliac crest; thus, patients with pathology at this level historically underwent consideration of posterior or true anterior (supine) approach surgery to address this level. Moreover, patients with ankylosed deformities, bony lateral recess stenosis, or previous retroperitoneal scarring are not candidates for this procedure [9,10]. Ultimately, even if patients are otherwise excellent candidates for lateral lumbar interbody fusion, the inherent need for a position change from the lateral decubitus to prone for pedicle screw placement, posterior decompression, or osteotomies introduces inefficiencies that may dissuade surgeons from adoption.

To help mitigate the inefficiency of intraoperative position changes, surgeons began performing percutaneous pedicle screw fixation in the lateral decubitus position. This eliminated the necessity to rotate the patient and change bed types. The placing of percutaneous pedicle screws in the lateral position has its own set of nuances, however, including ergonomic and spatial difficulties while the fluoroscopy unit is in the anterior–posterior orientation as well as difficulties placing the “down-side” screws. The introduction of robotics and CT-navigation helped to mitigate several of these issues, and these techniques have been well described as having excellent safety profiles [11,12]. Furthermore, the introduction of the oblique (OLIF) and lateral-position anterior lumbar interbody fusion (lateral ALIF) broadened the versatility of traditional single-position lateral lumbar interbody fusion by providing surgeons with the ability to simultaneously address pathology at L5-S1. Clinical and radiographic outcomes following these surgeries are at least similar, if not improved, compared to traditional anterior–posterior surgery [13,14,15].

Despite these advancements, lateral single-position surgery is still limited to indirect decompression. For patients requiring additional osteotomies or direct decompression, the prone position is still necessary for this additional bony work. Furthermore, the lateral decubitus position does not maximize lumbar lordosis [16]. Additionally, finding and exchanging the previous instrumentation can be particularly challenging in the lateral position.

Thus, performing a lateral interbody fusion in the prone position would theoretically maximize the benefits of the familiarity and consistency of the prone position while also maximizing the utility of the lateral interbody approach. This approach would maintain the efficiency profile of single-position lateral decubitus surgery by removing the need for an intra-operative bed change. Additionally, an assistant could perform posterior exposure and potentially instrumentation removal while the primary surgeon completed the lateral interbody, or the assistant surgeon could close the lateral incision while the primary surgeon performed posterior instrumentation or additional osteotomies. Further theorized benefits included the downward displacement of the peritoneal contents and kidneys, as well as favorable changes to the psoas muscle and lumbar plexus anatomy.

This ushered in a new era of the prone transpsoas (PTP) approach to the lumbar spine. While this surgery is now performed internationally, there is still a relative lack of research summarizing its optimum indications and subsequent clinical and radiographic outcomes. As such, the authors aim to provide readers with a scoping review of prone transpsoas fusion.

## 2. Techniques

**Figure 1:**
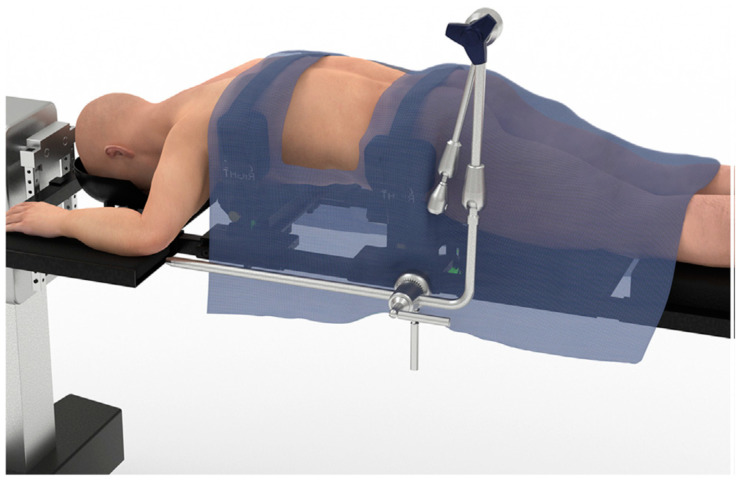
Depicts a patient laying prone on an open Jackson frame in the ‘Superman’ position with their arms extended. Around the chest and hips of the patient, affixed to the Jackson frame, is a custom PTP bolster (Alphatec Spine, Carlsbad, CA, USA). Each of the attachment points allows for independent coronal bending which facilitates greater access to lower lumbar segments (chest bolster rotated clockwise, hip bolster rotated counterclockwise). Reprinted from World Neurosurgery, Volume 149, Tyler G Smith, John Pollina, Samuel A Joseph Jr, Kelli M Howell, Effects of Surgical Positioning on L4-L5 Accessibility and Lumbar Lordosis in Lateral Transpsoas Lumbar Interbody Fusion: A Comparison of Prone and Lateral Decubitus in Asymptomatic Adults, e705–e713, Copyright (2021), with permission from Elsevier [18].

First described by Luiz Pimenta and William Taylor, the surgical technique for the PTP approach begins with the induction of general anesthesia, placement of foley catheter, and neurophysiologic monitoring [17]. The patient is then turned prone into an open Jackson frame, with the arms in the “superman” position. The patient is then secured using tape, or straps, around the chest and hips. The lateral flank and back are then prepped and draped in the standard fashion. The surgeon may elect to outfit the Jackson table with a custom PTP bolster, which allows for coronal bending at the hip and facilitates better L4-5 access, as shown in Figure 1 [18].

Markings are made on the skin to define key anatomical landmarks, including the anterior and posterior borders of the target disc space. In PTP, the skin incision favors the posterior aspect of the target disc spaces—this is intentional, to counteract gravitational forces that often lead to ventral retractor migration. Retroperitoneal access begins with a sharp lateral skin incision and electrocautery-assisted dissection of the overlying musculature. Muscle layers are then carefully released using a reverse-scissoring technique until access to the retroperitoneal space is achieved. A second incision in the retroperitoneum is typically not required, as the prone position draws the peritoneum far from the incision and access corridor. Next, blunt finger dissection is used to dissect through the retroperitoneal fat until the psoas muscle can be directly palpated. An initial dilator is then advanced using a surgeon’s finger. The psoas is then traversed using triggered EMG and fluoroscopy for guidance until the dilator has reached and docked onto the target intervertebral disc. The ideal docking position is dependent on the level and the vendor system. Once the position of the dilator is confirmed, a K-wire is inserted into the disc space to hold this position.

Next, circumferential EMG-stimulation-guided serial dilation is performed in proximity to the lumbar plexus. The authors use an 8 mA threshold for retractor placement, although this threshold varies widely amongst surgeons. The retractor is then advanced over the last dilator and expanded along the plane of the disc space. Finally, retractor placement is confirmed with fluoroscopy. Monopolar stimulation ensures the absence of neural tissue within the retractor blades and can serve as a positive control when it occurs dorsally beneath the blades. The K-wire is removed, and disc preparation is performed in standard fashion according to surgeon’s preference. Trial spacers and interbody placement are then performed. Once the implant is confirmed to be in a satisfactory location, the retractor is removed. Standard LLIF closure, which follows retractor removal, concludes the lateral portion of this procedure.

### 2.1. CT Navigation and Robotic Assistance

Several institutions have described their experiences with CT navigation and robotic-assisted prone-lateral surgery [12,19]. Compared to fluoroscopy, three-dimensional CT-guided navigation provides several key advantages. Notably, CT spinal navigation has repeatedly demonstrated decreased radiation exposure to the operative team while increasing surgical precision [20,21]. While there are limited data on its usage in PTP, the preliminary data demonstrate clinical feasibility and promising early results.

Positioning and draping for robotic- or CT-navigation assisted PTP is similar to that for standard fluoroscopy. Prior to the start of the surgery, a reference array may be docked to the posterior superior iliac spine via an iliac pin. Intraoperative CT or fluoroscopy-based robotic registration can then be completed, depending on the vendor. Skin marking can then be completed with navigated probes. For robotic-assisted PTP, the retractor is attached to the end-effector arm, and then guided and fixed into position. Subsequent disc preparation and trials may then proceed with navigational assistance. Care must be taken to account for changes in patient anatomy as a result of interbody placement and indirect decompression.

### 2.2. Fluoroscopic—Instrument Tracking

Novel fluoroscopy-based instrument tracking systems (TrackX, Hillsborough, NC, USA) have also been applied to prone transpsoas fusion. This combines several principles from standard fluoroscopy and CT-based navigation and provides an alternative to their associated weaknesses.

The patient is positioned and draped in a similar fashion to PTP performed with standard fluoroscopy. Instead of placing a reference array in the posterior superior iliac spine, a reference array is placed on a Jamshidi and malleted into the pedicle of an involved vertebral body (i.e., L4 pedicle for an L3-5 PTP). This allows for increased accuracy during the discectomy portion of the surgery, which often results in significant patient motion. Reference snaps are placed on the shafts of discectomy instruments, which are then calibrated to ensure accuracy. There are no intraoperative CT or registration scans. Instead, each instrument can be tracked on the surgeon’s screen using pseudo-live fluoroscopy, with confirmation and recalibration of accuracy performed with every subsequent X-ray. The discectomy and placement of interbody graft are then completed with tracking assistance. This technique has previously been described by Soliman et al. [22].

### 2.3. Case Example

Below, we present a representative case of PTP interbody fusion at our institution.

**Figure 2:**
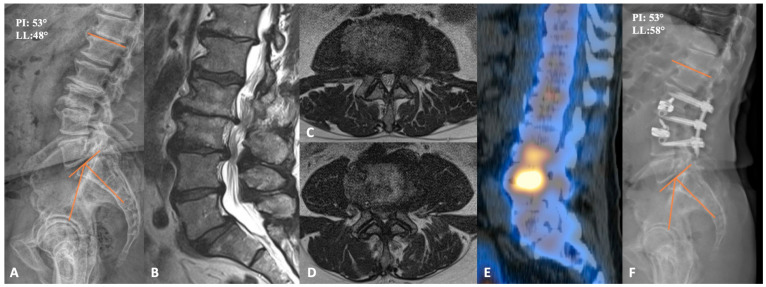
(**A**) preoperative lateral lumbar X-ray demonstrating advanced degenerative changes at L2-3 and L3-4. (**B**) sagittal T2-weighted MRI, together with axial cuts at L2-3 and L3-4 (**C**,**D**), respectively, showing severe central and bilateral lateral recess stenosis. (**E**) sagittal CT SPECT image demonstrating increased radiotracer activity at L2-4. (**F**) lateral lumbar X-ray of the postoperative construct with improved disc heights and lordosis. PI = Pelvic incidence; LL = Lumbar lordosis.

This patient was a 72-year-old-male who originally presented complaining of bilateral lateral thigh, hamstring, and low back pain for more than a year. Prior to presentation, he trialed physical therapy and epidural steroid injections at L2-3 and L3-4, which provided him with short-term pain relief. A lower-extremity neurologic exam was bilaterally unremarkable. Preoperative imaging demonstrated severe central canal stenosis at L3-L4, severe multilevel lateral-recess stenosis at L2-L3, L3-L4, and L5-S1 bilaterally, and grade 1 anterolisthesis of L5 on S1 without dynamic instability, as shown in Figure 2. CT-SPECT was also used to help understand the major pain generators. Increased uptake was seen around the disc spaces at L2-L3 and L3-L4. Importantly, there was no increased uptake in L5-S1, so it was felt that static spondylolisthesis was not a major pain generator. As the patient’s symptoms responded well to steroid injection, we decided to proceed with L2-4 PTP and posterior percutaneous fixation. The procedure was completed uneventfully in under 3 h, with a total of 100 mL blood loss. Postoperatively, the patient was discharged home on post-operative day 2 with resolution of bilateral lower-limb symptoms. Notably, lumbar lordosis increased from 48 degrees to 58 degrees, and his lower back pain gradually improved upon serial post-operative visit evaluations.

## 3. Evidence

### 3.1. Gain in Lordosis

Intraoperative prone positioning alone, facilitated by an open Jackson-frame, consistently demonstrates a notable increase in lumbar lordosis, ranging from 2 to 10 degrees when compared to upright and lateral decubitus positioning [18,23,24]. However, this positional advantage is not fully realized in traditional TLIF procedures, resulting in only modest post-operative lumbar lordosis gains when compared to lateral and anterior approach alternatives [24]. This difference is often attributed to the placement of a more lordotic interbody device via the transpsoas approach, facilitated by lateral access [24]. In a recent retrospective study involving 78 single-position lateral fusions, researchers reported lumbar and segmental lordosis gains roughly equivalent to half of the implanted cage lordosis [25]. Although this is a commendable advancement with regard to spinopelvic parameter improvement, we will demonstrate that PTP surgery can capitalize on both the lordosis gains facilitated by lateral interbody placement and the inherent increase in lordosis associated with the prone position.

In one large case series demonstrating the correcting ability of PTP surgery, Wang et al. reported average lumbar lordosis and segmental lordosis gains of 10.3 degrees and 10.1 degrees, respectively [26]. Notably, this team did not perform any posterior osteotomies and achieved average correction angles on the order of their chosen interbody, 10 degrees. Thus, combining the lordotic footprint of a lateral interbody fusion with the natural gain in lordosis from prone positioning is the ideal way to maximize lordosis. Further demonstrating this are several recent studies directly comparing PTP to other lateral or prone interbody fusion means where the PTP group consistently achieved greater lumbar lordosis [16,27,28,29].

Compared to TLIF in a propensity score-matched radiographic analysis, Soliman et al. found that PTP significantly improved lumbar lordosis angle (11.5 degrees versus 0.1 degrees), pelvic tilt (3.0 degrees versus −2.4 degrees), and pelvic incidence minus lumbar lordosis (15.5 versus 3.8) [28].

In several similar studies, researchers demonstrated statistically significant improvements in segmental lordosis for PTP compared to traditional lateral interbody fusion [16,27]. In a propensity-matched study of 18 patient pairs comparing arthrodesis in the traditional lateral decubitus position to the prone-transpsoas position, Amaral et al. reported an average segmental lordosis correction of 1.9 degrees in the lateral decubitus group and 6.6 degrees in the prone-transpsoas group (*p* = 0.02) [16]. Mills et al. quoted 14.8 degrees versus 6.6 degrees (*p* < 0.001) of post-operative lumbar lordosis improvement when comparing prone lateral to traditional lateral surgery [27]. Notably, these improvements were maintained at one-year follow-up.

### 3.2. Retroperitoneal Space and Major Vessels

**Figure 3:**
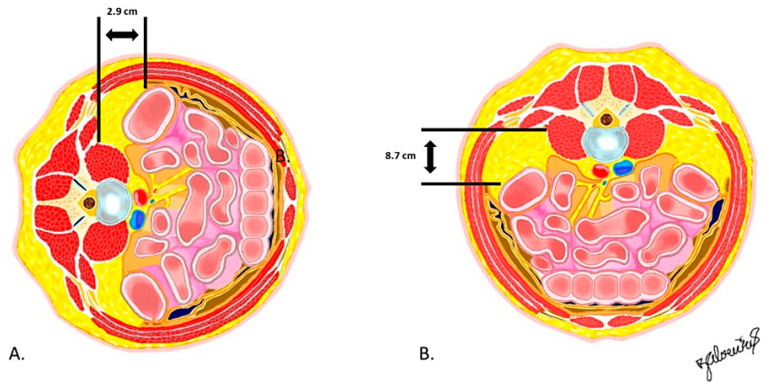
Axial slice at the lumbar spine, lateral (**A**) and prone (**B**), showcasing the larger corridor afforded in the prone position. Reproduced with permission from Dr. Luiz Pimenta, Risk of Injury to Retroperitoneal structures in Prone and Lateral Decubitus Transpsoas Approaches to Lumbar Interbody Fusion: A Pilot Cadaveric Anatomical Study; published by Cureus, 2023 [30].

The prone position pulls the peritoneum and ureter into a more ventral position; in a recent cadaveric study comparing risk of injury to retroperitoneal structures in the prone versus lateral positions, Pimenta et al. demonstrated less peritoneal violation and increased distance from the quadratus lumborum to the peritoneum in the prone cohort, as shown in Figure 3 [30]. Similarly, radiographic studies characterizing lumbar vascular anatomy describe surgically favorable shifts in the prone position. In a study of 30 volunteers without spinal deformity, Vaccaro et al. found that the iliac vessels were 3 mm more anterior to L4-S1 disc spaces [31]. Similarly, in severely kyphotic ankylosing spondylitis patients, Feng et al. reported that, from T9-L3, the aorta shifted laterally; below this level, the aorta migrated back over the midline, far from the disc space access [32]. Of note, both studies reported high anatomic variability [31,32]. Clearly, prone positioning provides several favorable anatomic advantages with regard to access trajectory; later, we describe how these changes are clinically translatable.

### 3.3. Psoas Anatomy and Lumbar Plexus

Several recent cadaveric and radiographic studies defining psoas and psoas-adjacent neurovasculature in the prone position suggest that the prone position is advantageous for access in several ways. Prone positioning draws the psoas muscle and femoral nerve posteriorly, providing greater distance from the middle of the disc space to critical branches of the lumbar plexus [33]. In turn, this wider corridor to the target operative site results in decreased traction along the lumbar plexus. This may theoretically reduce the likelihood of postoperative femoral plexopathy.

### 3.4. Efficiency

Single-position lateral spine surgery has been proven to improve surgical efficiency compared to traditional two-position counterparts. The advantages conferred by these operative time savings are multifocal and include a decreased risk of infection, decreased cost, and decreased risk of deep venous thrombosis [34]. Similarly, in the case of spine surgery, increasing operative duration was shown to increase post-operative medical complications, surgical complications, and risk of reoperation [35,36]. In fact, in a study of nearly 5000 patients, Hersey et al. found that each hour increase in operative time increased the risk of thromboembolism, transfusion, and total postoperative complications [37]. Furthermore, these time savings have a direct financial impact, as some studies have showed a per-minute operating room cost of 93 USD in image-guided spine surgery [38]. PTP access spine surgery utilizes the single-position concept and allows two surgeons to work simultaneously, maximizing operative efficiency.

Differences in operative times for traditional two-position surgery and lateral-only surgery are well documented. Time savings ranging from 35 to 157 min, while maintaining a similar or better complication profile, have been reported in studies comparing single- to two-position surgery [13,27,39,40,41,42,43,44]. Comparatively, studies detailing operative time for prone transpsoas surgery are scant and limited to several small retrospective cohort studies. Although sparse, the available literature describing these time savings is consistent and robust. In a retrospective cohort study providing a direct comparison between PTP and lateral two-position surgery, Buckland et al. and Lamartina at al. reported PTP operative times of 151 and 133 min versus two-position times of 206 and 182 min, respectively—time savings of approximately 50 min in both cases [13,45]. Much of the afforded increase in operative efficiency may be attributed to avoiding patient repositioning procedures, which are variable and inefficient across institutions.

Finally, we expect the operative time-savings to increase as adoption and familiarity increase. In a recent retrospective study, Patel et al. reported that their psoas retractor time decreased from 38 min to 9 min across their first 92 cases, demonstrating a remarkable learning curve [46]. Importantly, during the implementation period, the complication rate did not exceed the national reported ranges for two-position surgery. Taken together, the demonstrated operating room efficiency increases, the further increase in efficiency expected as surgeon proficiency improves, and the negative sequalae of increased operating room time suggest that PTP procedures hold potential for optimizing surgical course without compromising safety. It should be noted from the discussion above that the PTP approach is not simply a lateral decubitus approach in the prone position. The changes in anatomy and the effects of gravity are real, and it is advised that surgeons, even if comfortable in the traditional lateral decubitus position, undertake special training before attempting this surgical approach.

### 3.5. Limitations

One of the major limitations of PTP is the inability to address L5-S1 pathology. In the case of pure lateral decubitus surgery, this was solved with the advent of the lateral ALIF at L5-S1. Currently, the solution is pure supine surgery at L5-S1, before flipping to PTP and posterior work. There is some controversy regarding whether lateral ALIF at L5-S1 affords the same visibility and correction of segmental lordosis as compared to supine ALIF; however, early reports suggest it is at least as safe, with commendable spinopelvic parameter improvements [47,48]. Given the favorable anatomic changes in the prone position, as described in Section 3.1, further study is required to more completely inform recommendations for optimal L5-S1 access. Another possible limitation of PTP is how to deal with thoracolumbar junctional pathologies. Methods to deal with the diaphragm and great vessels have been determined in the lateral decubitus position, but it is unclear whether the same methods can be transferred to PTP [49].

## 4. Complication Profile

As an emerging surgical technique with known and well-established alternatives, it is important to characterize the complication profile of prone-transpsoas access-spine surgery early and often to ensure the benefits are conferred while maintaining an acceptable safety profile. To date, in cases where prone lateral surgery is undertaken, its novelty has precluded a large number of studies, but the recent literature from Farber et al., Soliman et al., and pooled analyses have shown that complications, of all causes, are comparable to those in conventional, two-position, lateral surgery [50,51]. Likewise, looking at individual complications, it has been demonstrated that critical complications, including post-operative neurologic deficit, inadvertent anterior longitudinal ligament release, vascular or ureter injury, spinal fluid leak, and psoas hematoma, are comparable or decreased in relation to traditional lateral surgery.

### 4.1. Neuropathy

The most common complications arising from both prone lateral and traditional lateral access spine surgeries are new post-operative sensory and motor deficits. Defects include ipsilateral paresthesias, dysthesias, and lower-extremity weakness. In a systematic review examining thigh symptoms after traditional lateral interbody fusion, Gammal et al. reported an incidence range for new-onset postoperative sensory changes at 3.1–60.7% [52]. Authors cite inconsistent reporting standards for this wide range, stating that many authors of the included studies view transient neurologic symptoms as side-effects of required psoas manipulation rather than a true complication. Comparatively, in a 365 PTP-patient retrospective study by Soliman et al., the authors showed that prone lateral fusion resulted in new onset radiculopathy or lower-extremity sensory symptoms in only 8.2% of patients [51]. Farber et al. and Patel et al. reported similarly low rates of new onset of thigh neuropraxia among their PTP cohorts, at 13.3% and 11.9% respectively [46,50].

With respect to motor deficits in lateral position surgery, Hijji et al., in a meta-analysis of nearly 7000 patients, cited an incidence of 14.1% for new onset transient motor weakness and 5.1% for permanent motor or sensory loss [5]. Comparatively, in Soliman et al.’s multi-center PTP study, only 21 of 365 (5.8%) patients developed ipsilateral motor weakness—5 (1.3%) of which were persistent after 7-month follow-up [51]. Likewise, Farber et al. and Patel et al. reported few long-term post-operative motor deficits during one-year follow up, 1.2% and 0%, in their respective retrospective studies [46,50].

Although these data on prone lateral surgery are limited in sample size, the current evidence suggests that prone lateral surgery is safer than traditional lateral decubitus access with respect to new onset sensory and motor deficits. Taken together with the previously described cadaveric and radiographic studies describing more favorable access anatomy, the prone position anatomic augmentations are clinically translatable.

### 4.2. Inadvertent Anterior Longitudinal Ligament Release

In the aforementioned lateral–decubitus meta-analysis by Hijji et al., the rate of inadvertent anterior longitudinal ligament (ALL) release was reported to be 2.9% [5]. Similarly, in their PTP cohort, Soliman et al. reported an ALL rupture incidence of 2.2%, totaling 8 of the 365 patients [51]. Farber et al. (5, 2.3%) and Buckland et al. (3, 1.2%) describe similar rates of ALL release in their PTP cohorts [13,50]. Further, in their single-surgeon, retrospective, prone lateral interbody fusion study, Patel et al. reported that all-cause complications, including ALL rupture, occurred only in the first 20% of patients, suggesting an inherent learning curve [46]. It is expected that increased repetitions will lead to the identification and avoidance of intraoperative factors such as gravity-induced ventral retractor migration.

### 4.3. Vascular Injury

Vascular injury is a dreaded, but exceedingly rare, complication of spinal surgery and associated access. Hijji et al. cited a single (0.12%) aortic injury and two (0.25%) common iliac vein lacerations in their meta-analysis of lateral interbody fusions [5]. Similarly, Soliman et al. cited the incidence of major vascular injury, common iliac vein laceration, at 1 (0.3%) in their multicenter study [51]. Notably, this occurred during ALIF access, not the prone-transpsoas portion of the procedure. Farber et al. described five cases (2.0%) of segmental artery bleeding in their systematic review, all of which arose from the same study and were controlled intraoperatively with no sequalae [50]. There were no universal reporting standards for vascular injuries in these studies; it may be the case that minor vascular injury, including controlled segmental bleeding, is largely underreported. The favorable neurovascular shifts while prone and the larger safe zone created by prone positioning may led to less blood loss in this position; however, this requires further study [30,53,54].

### 4.4. Other (Durotomy, Hematoma, and Ureter Injury)

The last of the major prone-transpsoas surgery complications, durotomy, psoas hematoma, and ureter injury, have a comparable or better cited incidence than those described in traditional lateral surgery. Hijji et al. reported durotomy, psoas hematoma, and ureter injury rates of 1.8%, 1.1%, and 0.9%, respectively [5]. Comparatively, Farber et al. (F) and Soliman et al. (S) reported a similar or lower incidence of these feared complications—durotomy: 0.6% (F) and 0% (S), psoas hematoma: 1.3% (F) and 0.5% (S), and ureter injury: 0.3% (S) [50,51].

When evaluating the existing data within the published literature, it becomes evident that the emerging prone lateral technique exhibits a commendable safety record and acceptable complication profile. It is critical to acknowledge that all the studies on PTP published to date have a low level of evidence and are limited to retrospective case series. Additionally, it is important to note that the comparisons made to traditional lateral surgery are not direct comparisons with respect to patient selection and confounders; therefore, medical decisions cannot be made from these data alone. However, when synthesizing the available literature, it is clear that prone lateral surgery is at least as safe as its longer-standing counterparts. To gain a more comprehensive understanding of the long-term complication rates associated with this approach, further research involving extended follow-up and randomized prospective study is imperative.

## 5. Current Applications

The transpsoas approach is gaining traction in modern spine surgery due to its versatility in addressing various conditions, including degeneration, adjacent segment disease, deformity, anterior column realignment, corpectomy, and pseudarthrosis. This novel access trajectory combines the anatomic advantages of both prone and lateral surgery, without compromising, and in many cases improving, patient safety and outcomes, and without necessitating patient repositioning.

Only recently have lumbar corpectomies, anterior column realignments, and deformity corrections been performed via a lateral decubitus transpsoas approach. Lateral decubitus positioning confers several key advantages in these cases compared to the prone counterpart, such as the improved biomechanical stability imparted by larger interbody cages when combined with posterior fusion [24]. The lateral decubitus position alone, however, does not facilitate complex cases involving trauma, deformities, or large tumors, which require multi-stage surgeries for direct, open, posterior column access. Taken together, in cases where lateral, transpsoas access facilitates key advantages and open posterior access is required, PTP surgery allows for the maintenance of operative efficiency without compromise.

### 5.1. Degenerative Spondylolisthesis

As PTP adoption increases, the literature has begun to demonstrate wide-ranging clinical applications that significantly overlap with the conditions currently treated with two-position surgery. In the case of spondylolisthesis, the gold standard treatment is decompression and fusion, as well as circumferential surgery [55,56]. Similarly, in the case of adult degenerative scoliosis, surgical treatment options range from neural decompression alone to decompression plus arthrodesis [57]. Although there is a paucity of studies describing PTP for de novo degenerative spondylolisthesis, a case series by Stone et al. describes several challenging degenerative cases that were aided by this access trajectory, including grade-1 spondylolisthesis, coronal deformity, and degenerative scoliosis [58]. The advantage of PTP in these cases is clear: the posterior column is accessed in the more familiar and anatomically favorable prone position, neural elements are decompressed, and the spinal column is stabilized without patient repositioning.

**Figure 4:**
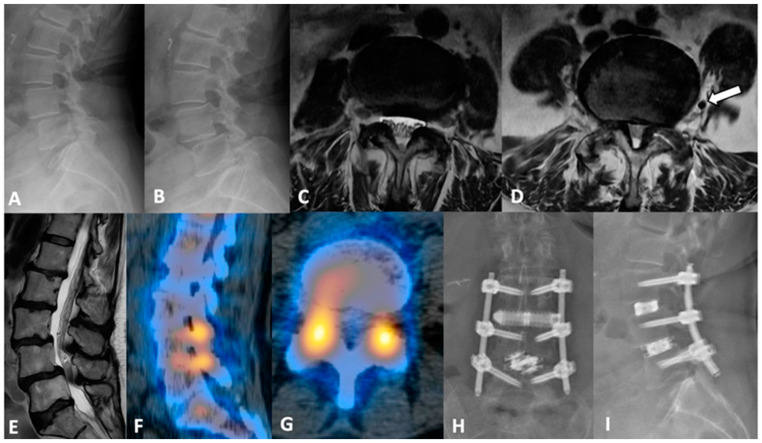
(**A**,**B**) dynamic lumbar films with variable grades of spondylolisthesis at L3-4 and L4-5. (**C**) axial MRI view at the L3-4 level, and (**D**) at L4-5 level with (white arrow) a sizable vessel in the left lateral space. Psoas muscle morphology and considerable canal stenosis are showcased via (**E**) sagittal view. (**F**,**G**) sagittal and axial CT SPECT slices demonstrating increased uptake at both L3-4 and L4-5 facets, confirming pain location. (**H**,**I**) postoperative AP and lateral lumbar films.

The authors present a representative case highlighting the correcting power of PTP, as well as its ability to be combined with other posterior approaches as needed. Case 2 is a 70-year-old-female who originally presented with lower back pain radiating to the right leg and grade 4+/5 weakness on ankle dorsiflexion. Prior to presentation, she exhausted all non-surgical treatment modalities. Preoperative imaging was notable for grade 1 spondylolisthesis at L3-4, dynamic instability affecting L4-5, and evidence of severe central stenosis and bilateral foraminal stenosis of L4-5. CT SPECT revealed an increased radiotracer uptake at both levels’ facet joints. Importantly, there appeared to be a vessel at the mid-point of the vertebral body at L4-5 (Figure 4D). Taking the unfavorable vascular anatomy hindering anterolateral access to the L4-5 disc space into consideration, we ultimately proceeded with L3-4 PTP interbody fusion and L4-5 TLIF. Notably, this procedure was completed without intraoperative complication. Postoperatively, the patient’s radiculopathy was improved, as well as her back pain. The patient was discharged home after 3 days.

### 5.2. Adjacent Segment Disease

Adjacent segment disease (ASD) is a well-described sequalae of interbody fusion that often requires surgical correction [59]. Single-position lateral decubitus surgery for ASD facilitates large interbody cage placement; however, it requires posterior instrumentation to achieve optimal construct stability [24,60]. These ASD cases, which require instrument exchange and direct posterior decompression, require an intraoperative position change. PTP has gained traction in this avenue as it offers a means of harnessing the advantages of lateral access in prone positioning. The evidence on PTP for ASD is limited; however, in the largest case series to date, Wang et al. demonstrated the feasibility of PTP in ASD intervention without compromising safety [26]. Again, this team was able to achieve lordosis correction matching their chosen interbody of 10 degrees without posterior osteotomy [26].

**Figure 5:**
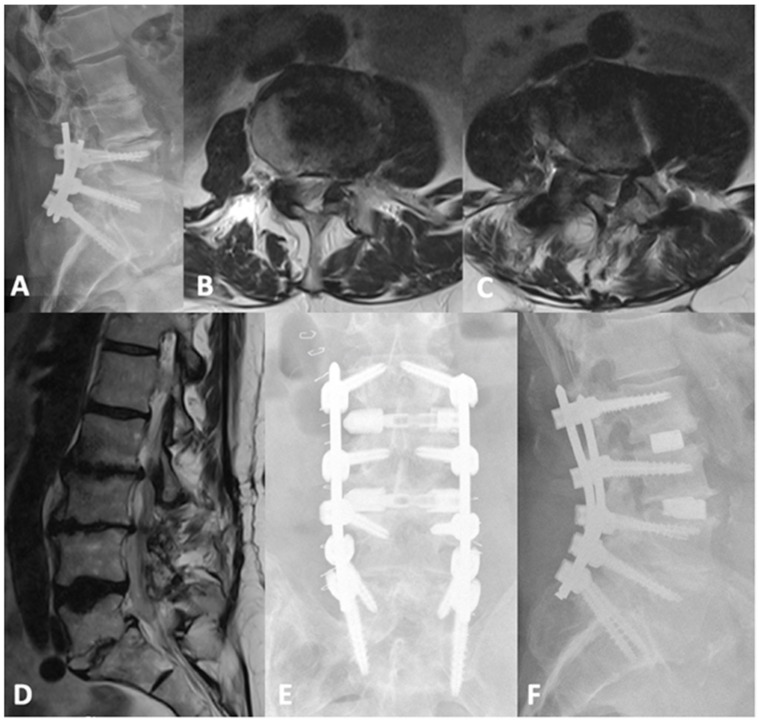
(**A**) lateral lumbar X-rays of the previous L4-S1 fusion with advanced degenerative changes and decreased disc heights at L2-3 and L3-4. Axial T2-weighted MRI sections of (**B**) L2-3 and (**C**) L3-4 levels and (**D**) a sagittal slice demonstrating the bilateral recess and central canal stenosis. (**E**,**F**) are postoperative AP and lateral views showing the extension of the fusion to L2 and augmented disc heights.

Below, the authors present a representative adjacent segment disease case. Case 3 is an 82-year-old male with remote history of L4-S1 minimally invasive decompression and fusion for back pain. Three years later, he re-presented with recurrent low back pain and intermittent radiation to both lower limbs. New imaging showed L2-L3 and L3-L4 moderate-to-severe spinal canal stenosis and moderate bilateral neuroforaminal narrowing. Since the offered L2-3 and L3-4 facet injections provided him with adequate but temporary pain relief and the evidence of ASD, we ultimately offered and proceeded with L2-4 PTP interbody fusion with extension of previous posterior instrumentation. Preoperative pelvic incidence (PI) and lumbar lordosis (LL) were 54° and 47°, respectively. LL improved to 56° postoperatively. The surgery was completed with 200 mL estimated blood loss, and without complication. The postoperative course was uneventful, and the patient was discharged home, Figure 5.

## 6. Complex Applications

### 6.1. Deformity

As is the case with other spine pathologies, deformity is often a quality-of-life-impacting condition [61]. Definitive surgical correction is often required; however, these procedures are typically large, open cases associated with significant risks and complications [62]. Minimally invasive surgery such as LLIF and its variations are effective means of deformity correction in many cases and mitigate many of the pitfalls of open spine surgery [63]. Staged or two- or three-position surgeries paved the way for severe deformity correction necessitating open posterior access; however, patient repositioning and the increased time under general anesthesia also lead to the previously described increased morbidity. PTP surgery represents the logical progression in access trajectory and technique advancements; early reports of PTP-based deformity correction highlight its utility.

We have started to develop experience of using PTP in deformity correction. Our preliminary results suggest that, with PTP alone, the correction angles were not on the order of those achievable by anterior column realignment (ACR) or pedicle subtraction osteotomy (PSO), but the potentially deformity-correcting benefits of PTP are two-fold: (1) PTP allows for simultaneous posterior instrumentation, which will theoretically allow for a greater degree of correction by complementing PTP with more invasive correcting procedures such as osteotomies, and (2) less severe deformities, as in MISDEF 1-3, may be successfully treated by less invasive means [26].

**Figure 6:**
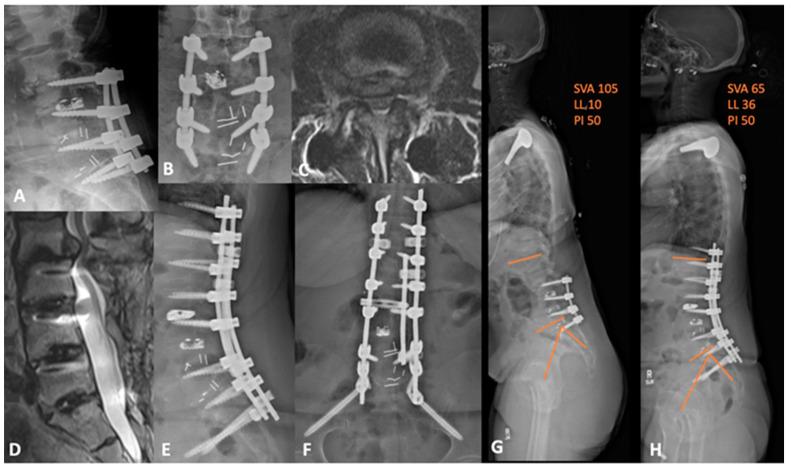
(**A**,**B**) preoperative lateral and AP lumbar X-rays demonstrating the advanced degenerative changes at L2-3 with grade 2 anterolisthesis. (**C**) axial and (**D**) sagittal T2-weighted MRI showing the adjacent segment disease at L2-3 with severe central and lateral recess stenosis. (**E**,**F**) lateral and AP lumbar X-rays of the postoperative construct with apparent deformity correction. (**G**) preoperative and (**H**) postoperative whole-spine films demonstrating an improvement in sagittal balance and global lordosis. SVA = sagittal vertical axis; LL = lumbar lordosis; PI = pelvic incidence.

Below, the authors present two representative deformity cases. Case 4 is a 60-year-old female, with two previous operations, who was referred to us from another institution. She has a remote history of L3-4 decompressive laminectomies and, most recently, L3-S1 TLIF 3 years prior to presentation. She presented complaining of severe low back pain with radiation to both thighs anterolaterally. Her imaging was notable for L2-3 ASD, anterolisthesis, and positive sagittal imbalance with a PI-LL mismatch—flat-back deformity. We therefore decided on L2-3 PTP lateral interbody fusion, L2-3 posterior column osteotomy (PCO), L4 PSO, and T11-pelvis fusion extension. The postoperative course was uneventful, and the patient had substantial symptomatic relief with improved sagittal balance and a PI-LL mismatch of 14, compared to 40, preoperatively, as shown in Figure 6.

**Figure 7:**
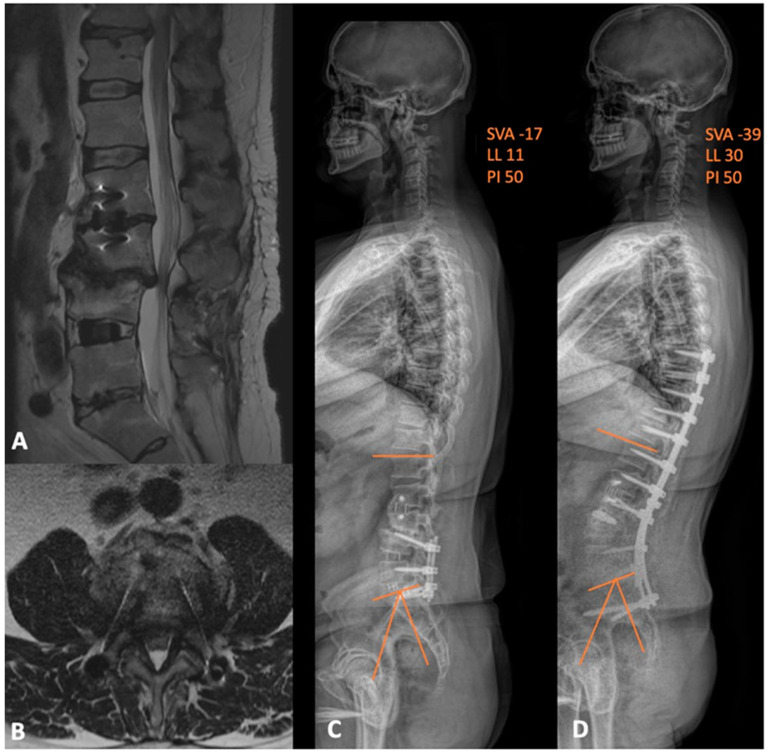
(**A**) sagittal and (**B**) axial MRI views depicting the adjacent segment disease at L3-4 level with resultant severe central and lateral-recess stenosis. (**C**) preoperative and (**D**) postoperative whole-spine films demonstrating the improvement in both the global lumbar lordosis and PI-LL mismatch. SVA = sagittal vertical axis; LL = lumbar lordosis; PI = pelvic incidence.

Case 5 is a 53-year-old male who was also referred to our institution with prior history of L4-S1 fusion and standalone L2-3 lateral interbody fusion at another institution. He was wheelchair-bound and complaining of severe lower back and left-sided leg pain that radiated to his foot. Prior to presentation, he exhausted all conservative options without symptomatic improvement. His new imaging showed central canal stenosis with severe, diffuse disc bulge, degenerative facet changes, and posterior subluxation of L3 on L4 with significant PI-LL mismatch. He also had evidence of thoracic stenosis, which was worst at T9-T10 and T10-T11. We proceeded with L3-4 PTP, L3-4 PCO, L4 PSO, and an extension of fusion from the T8-pelvis with thoracic decompression. The patient did well after surgery, with significant improvements in his preoperative symptoms and adequate correction of his PI-LL mismatch, as shown in Figure 7. Three months postoperatively, he began ambulating without assistance. This case is an excellent example of how single position surgery not only allows for maximized deformity corrections, but also facilitates other surgical goals, such as direct decompression.

### 6.2. Anterior Column Realignment

Traditional techniques to correct sagittal plane deformities include osteotomies, which require open posterior access. More recently, an anterior column, minimally invasive approach was developed, with demonstrated success in correcting sagittal plane misalignment by sectioning the anterior longitudinal ligament and placing a hyperlordotic cage, with anterior column realignment. Lateral minimally invasive anterior column realignment has repeatedly demonstrated superiority compared to traditional posterior osteotomy with respect to blood loss while maintaining a similar overall complication rate [64,65,66]. In the case of severe, sagittal, plane fixed deformities, however, minimally invasive lateral ACR is not as effective as open posterior surgery. Combining the two methods in a hybrid approach can maximize their benefits—pedicle subtraction osteotomy optimizes achievable segmental lordosis at the adjacent ACR level [67]. Further, anterior support via interbody cage placement provides support for PSO by providing a significant reduction in rod stress; the effect is amplified with additional cage placements [68].

In cadaveric and clinical feasibility studies, this hybrid surgery resulted in a stepwise increase in lordotic correction, greater than that achieved by either procedure alone; however, these procedures were performed on separate days, depending on the length of the ACR [67]. Here, PTP provides the key advantage of eliminating the requirement for multi-stage surgery and the morbidity associated with the previously described decreased surgical efficiency.

These ACR-PSO hybrid surgeries are not without inherent drawbacks, and prone surgery is not without limitations in the case of ACR. One underreported and feared complication, with an unknown precise incidence, is great vessel violation. In these circumstances, the patient must be flipped supine or rotated to a lateral decubitus position to extend the skin incision for vascular repair. As previously described, some patients have a lumbar-vascular anatomy that is favorably augmented by the prone position. In these patients, perhaps the advantages afforded by the prone position, anatomic and surgical duration balance the risk of major vascular injury—appropriate patient selection is paramount.

### 6.3. Corpectomy

Vertebral corpectomy is a powerful surgical technique that may be deployed to treat complex pathologies, including traumatic spinal cord injury, cancer, infection, and wide-ranging deformity corrections. Pathology and surgeon preference dictate access, either posterior, anterior, or lateral, each with its respective advantages and disadvantages. The posterior approach offers a familiar route to spinal surgeons while the anterior approach provides superior direct visualization of the ventral spinal elements [69]. Combining the benefits from the anterior and posterior approaches, the lateral approach, traditionally in the lateral decubitus position, offers access to the anterior and lateral columns without damaging posterior musculature or requiring an access surgeon to navigate the peritoneal vasculature [69]. Less studied, however, are corpectomies via PTP. This novel approach combines the advantages of the above access trajectories while additionally gaining the lordotic anatomic advantages imparted by the prone position.

In complex corpectomy cases requiring complete circumferential access to the anterior and posterior columns, the PTP approach has recently demonstrated its utility in several case studies. Gandhi et al. described two corpectomy cases necessitating simultaneous lateral and posterior access to the spinal column [70]. Specifically, Gandhi and colleagues elected this approach to perform the more extensive decompression allowed in the prone position while maintaining the ability to manipulate the anterior column through lateral access [70]. In this study, the postoperative course was uneventful, and the patient successfully recovered [70]. As is the case with other PTP applications, and as demonstrated in this case, the surgical team maintains the ability to perform other indicated procedures that require prone positioning without flipping the patient; once again, operative efficiency is maintained.

### 6.4. Oncology

In the case of oncologic resections, the larger safe zone imparted by both gravitational forces and the ability to extend the hips may theoretically allow for more tissue-sparing resections due to the better and more direct visualization of cancerous masses. Gandhi et al. described a case of a solitary plasmacytoma requiring aggressive local control and spinal column height restoration [70]. Here, a PTP approach facilitated simultaneous posterior and lateral access for resection, direct posterior decompression, and the larger lateral interbody placement allowed via transpsoas access.

### 6.5. Pseudoarthrosis and Implant Retreival

Pseudoarthrosis, like ASD, is a well-described morbidity of arthrodesis. Traditionally, pseudoarthrosis is treated with a different surgical approach than the index procedure and with the addition of other materials such as bone grafts and instrumentation. The use of interbody devices in pseudoarthrosis correction has shown higher fusion rates, approaching 100%, in circumferential surgeries where an interbody is placed laterally or anteriorly, and instruments are exchanged or placed posteriorly [71,72,73]. In such cases, where two-stage surgery has demonstrated superiority, the advantage imparted by PTP is clear; the patient and operating room staff may avoid the unnecessary repositioning procedures. Lastly, in these cases, which frequently require instrument exchange, the simultaneous dual access to the spinal column may be advantageous. A failed implant may be exchanged by simultaneous posterior manipulation and lateral retrieval. There is no description of such in the literature; however, simultaneous posterior and anterior column access may be advantageous in the appropriate clinical context and adapted for various scenarios.

## 7. Conclusions

PTP surgery is an emerging technique offering several advantages over its traditional two-position counterparts. The current body of evidence supports that PTP surgery can achieve equal or better lumbar lordosis, pelvic tilt, and segmental lordosis parameters as compared to traditional lateral interbody fusion or transforaminal lumbar interbody fusion. Anatomically, the prone position allows for greater access to the target operative site with less obstruction by nearby structures, resulting in the equal or better complication profile of PTP compared to conventional access means. Efficiency is a key advantage of PTP as it minimizes operative time, thereby reducing complication risk and operative cost. Finally, PTP has a wide range of applications. It allows for the advantages of lateral access while eliminating the need for patient repositioning, making it a versatile option for various disease processes. Although the literature supports the safety and efficacy of PTP, it is a new technique; prospective studies with randomization will be required to completely describe PTP and inform recommendations. Overall, PTP shows promise in revolutionizing spine surgery by offering improved efficiency and outcomes without compromising safety.
